# Patterns and Associated Factors of Caesarean Delivery Intention among Expectant Mothers in China: Implications from the Implementation of China’s New National Two-Child Policy

**DOI:** 10.3390/ijerph13070686

**Published:** 2016-07-07

**Authors:** Lianlian Wang, Xianglong Xu, Philip Baker, Chao Tong, Lei Zhang, Hongbo Qi, Yong Zhao

**Affiliations:** 1 The Department of Obstetrics, The First Affiliated Hospital of Chongqing Medical University, Chongqing 400016, China; llian_w@163.com (L.W.); chaotongcqmu@163.com (C.T.); 2 Department of Reproduction Health and Infertility, The First Affiliated Hospital of Chongqing Medical University, Chongqing 400016, China; 3 Canada-China-New Zealand Joint Laboratory of Maternal and Fetal Medicine, Chongqing Medical University, Chongqing 400016, China; philip.baker@leicester.ac.uk; 4 School of Public Health and Management, Chongqing Medical University, Chongqing 400016, China; xianglong1989@126.com; 5 Research Center for Medicine and Social Development, Chongqing Medical University, Chongqing 400016, China; 6 The Innovation Center for Social Risk Governance in Health, Chongqing Medical University, Chongqing 400016, China; 7 College of Medicine, Biological Sciences and Psychology, University of Leicester, Leicester 3182, UK; 8 Research Center for Public Health, School of Medicine, Tsinghua University, Beijing 100062, China; lzhang20@mail.tsinghua.edu.cn; 9 Central Clinical School, Faculty of Medicine, Nursing and Health Sciences, Monash University, Melbourne 3182, Australia; 10 School of Public Health and Preventive Medicine, Faculty of Medicine, Nursing and Health Sciences, Monash University, Melbourne 3053, Australia; 11 Melbourne Sexual Health Centre, Alfred Health, Melbourne 3053, Australia

**Keywords:** caesarean delivery intention, expectant mothers, two-child policy, China

## Abstract

*Objective*: This study explores the basic demographic characteristics of expectant mothers in the context of their intentions regarding mode of delivery, in particular, the preference for caesarean delivery, and analyzes the social and psychological factors that influence delivery preference. *Method*: A cross-sectional survey of pregnant women was conducted during June to August in 2015. This study adopted a stratified sampling method, and 16 representative hospitals in five provinces of China were included. *Results*: 1755 and 590 of expectant mothers in their first and second pregnancies, respectively, were enrolled in this study. 354 (15.10%) intended to deliver by caesarean section and 585 (24.95%) participants were uncertain prior to delivery. 156 (8.89%) of expectant mothers in their first pregnancy and 198 (33.56%) expectant mothers in their second pregnancy intended to deliver by caesarean section. Ordinal logistic regression analysis found that nationality, parity, trimester of pregnancy, and advanced maternal age were factors associated with intention to deliver by caesarean (ordered logistic regression/three-level caesarean delivery intention criterion; odds ratios *p* < 0.05). *Conclusions*: 8.89% of first pregnancy expectant mothers and 33.56% of second pregnancy expectant mothers intended to deliver by caesarean section. Any intervention program to reduce the rate of Caesarean delivery should focus on the Han population, older pregnant women, and expectant mothers in their second pregnancy, at an early gestation.

## 1. Introduction

In 1985, the World Health Organization declared in Fortaleza, Brazil, that “There is no justification for any region to have a caesarean section rate higher than 10%–15%” [1]. The statement was based on the good maternal and perinatal care outcomes of the Nordic countries. In middle/low-income countries, the optimal caesarean section (CS) rate is influenced by preferences regarding delivery, available medical services, family income, and the health care professionals’ qualifications, as well as parents’ education level. Nevertheless, CS rates show a significantly increasing trend worldwide [2]. The most likely reasons include: reluctance to undergo a vaginal delivery (particularly amongst pregnant women with previous CS experiences), little improvement in facilities for vaginal delivery, and increased antenatal indications for CS consequent on improved equipment and technology (such as 3D ultrasound, fetal monitoring, fetal mirror, etc.) [3].

There is a growing trend in China for CS, from 2.0% (14/701) in 1978–1985 to 36.6% (813/2224) during 2006–2010 [4]. A 2011 survey conducted in multiple regions of mainland China found that the CS rate was 54.5%; 24.6% of these were performed in the absence of any medical indication [5]. Reports from 2014 show CS rates in China of 54.90% [6], and 58.5% [7]. In the United States, CS has become the most common surgical procedure; the CS rate increased from 20.6% in 1997 to 31.5% in 2009 [8]. In recent years, the CS rate in Europe was 19%–33% [9], in South America rates were 30%–50% [10], and there is a rising tendency in Africa and other regions [11]. Molina et al., further investigated the CS rate for all WHO Member States; 45 countries had a CS rate ≤7.2%, 48 countries 7.2%–19.1%, 48 countries 19.1%–27.3%, and 53 countries >27.3% [3]. The increasing trend was illustrated by a significant average annual increase in primary (4.3%) and repeat (4.8%) CS rates from 1998 to 2008 in Australia [12].

Socio-economic factors have contributed to the increase in “unnecessary” CSs in China [7]. A large retrospective survey in China identified factors which led to CS delivery; these were not only personal (education, vocation, age of childbearing, residence) but also medical (for example hypertension, above average fetal weight, fetal malposition) [4]. The increased preference for CS is likely to be influenced by multiple additional factors, such as the financial status, underlying diseases, level of education, family/social environment, reproductive knowledge, media reports, feedback from social circles and medical staff, as well as the previous delivery experience [13]. Despite the greater number of complications and risks of CS as compared to vaginal delivery, women still tend to choose CS. Litorp’s investigation concluded that these women focused on a perceived benefit for their child, and overlooked the risk to themselves of going through a CS [14]. In respect of the women’s preference, health workers should also monitor the influence of different health care models [9].

In certain high-risk pregnancies, CS delivery is an effective measure to reduce maternal and prenatal mortality, however, high CS rates do not relate to any decline in maternal and neonatal mortality rates. Moreover, CS conveys risks of complications that are greater than those of vaginal delivery [15], (such as infant and maternal mortality, severe maternal complications (post-partum bleeding, organ damage, infection, pain, etc.). CS also triggers other socio-economic challenges, such as the excessive costs for infant intensive care, as well as for other health care resources. In Europe, it has been estimated that if appropriate decisions regarding mode of delivery were applied to those delivering after a previous CS, not only would 160,000 unnecessary caesarean sections be avoided annually, but about €150 million of additional expenses would be saved [16].

The increase in CS delivery over time has been maintained. As for the primipara, the increase may be related to differences in clinical decision making or maternal request. However, other reasons, such as anxiety, various pregnancy complications, a painful previous childbirth experience, as well as the neonatal morbidities caused by the vaginal delivery, may influence a choice to opt for CS [17]. On 29 October 2015, the Chinese government announced their new policy: an amendment to its 1978 single-child family policy would fully allow couples to have two children. Some expect that this may trigger the next baby boom in the mainland China. This study explores the socioeconomic status of the different populations on their preference for the caesarean sections. In particular, we compared the delivery preferences of women in their first and second pregnancies and analyze socioeconomic factors that impact on delivery preference.

## 2. Participants and Methods

### 2.1. Ethical Approval

All subjects gave their informed consent for inclusion before they participated in the study. The study protocol was approved by the Ethics Committee of Chongqing Medical University (record number 2015008).

### 2.2. Research Method

A cross-sectional analysis of expectant mothers from June to August in 2015 from 16 hospitals in five regions of Mainland China was undertaken. The sample consisted of pregnant women in five regions, namely, Chongqing, Chengdu, Zunyi, Liaocheng, and Tianjin, China. Chongqing, Chengdu, and Zunyi are in south China, Liaocheng and Tianjin are in north China. Face-to-face interviews using a survey questionnaire were conducted by the investigators who were specifically trained, medical students. The participants were categorized into two groups based on delivery times: Expectant Mothers in their First Pregnancy and Expectant Mothers in their Second Pregnancy.

### 2.3. Population and Sample

Participants were those pregnant women who want to get examined in obstetrics clinic. Data were stratified sampling selected pregnant women (both expectant mother of first and second pregnancy). In 2455 target interviewers, 55 participants declined to answer any questions, and the preliminary response rate was thus 97.76% (2400/2455). Among 2400 respondents, the final analysis sample included the 2345 persons who answered all questions.

### 2.4. Face Validation and Content Validity

The questionnaires were established by a panel of nine experts (three nutrition experts, two target population experts, two epidemiology experts and two health education experts).

### 2.5. Sampling Framework

This study was conducted in obstetrics clinic in selected hospitals. The following guidelines were implemented during the Hospitals survey. To reduce admission rate bias, hospital level was divided into Level 3A hospital, Level 2A hospitals, and Level 2B hospitals and below. According to the hospital level, we randomly sixteen hospitals in five regions of Mainland China were included in this study.

### 2.6. Survey Administration

#### 2.6.1. Participants—Pregnant Women Feeding Service Social Media-QQ Group

To better understand the needs of pregnant women and give better service for pregnant women, we established a social media communication group (QQ group). This QQ group conveniently provided research personnel to answer questions raised by pregnant women and facilitate communication between pregnant women and researchers. Our research group’s members answered pregnant women’s questions in a timely manner.

#### 2.6.2. Network Communication among Team Members

Network communication: we used a social media mobile app QQ (Tencent Group, Shenzhen, China) to strengthen communication among team members. We promptly collated and shared problems encountered during the investigation through the group, in order to exchange knowledge and experience. The network was used to share relevant data, and to provide online training for investigators.

#### 2.6.3. Investigators

Team members were from different grades, including undergraduate and graduate students, academics from Chongqing Medical University, Chengdu University of Traditional Chinese Medicine, Jackson State University and the University of Adelaide, and hospital obstetricians. All investigators underwent standardized training and were familiar with the objectives and methodology.

#### 2.6.4. Process of Development of Questionnaire

We designed and modified the questionnaire as follows. First, after the first draft of the questionnaire, students met to modify the questionnaire. Second, teachers from School of Public Health and Management, Chongqing Medical University modified the questionnaire. Third, Invited obstetricians revised the questionnaire. Fourth, foreign experts modified the questionnaire.

#### 2.6.5. Pilot Study

A total of 24 individuals participated in a pilot test in June 2015. The questionnaire was subsequently modified, according to results of the pilot. Also, it became apparent that investigators required further training; this was provided.

#### 2.6.6. Work Manual of Investigators

The manual was divided into two parts. The first part contained the overall plan; it included the background, purpose, technical route, research method, data processing, etc. It was written in the relatively simple language, to ensure that it was understood by all of the students. The second part contained details regarding organization and implementation, including the preparation of our materials, application of various funds, progress in other work, and our modification consequent on the pilot test. In this section, some of the important aspects of the investigation were stressed, to avoid mistakes during the investigation, and also to add relevant literature concerning the interview skills. We delivered the work manual to each member of the research group.

#### 2.6.7. Questionnaire

The questionnaire was customized for the target population, with modifications based on the pilot study. The final draft of the questionnaire was agreed after several discussions with experts after review of the pilot investigation. We modified the questionnaire, especially the presentation of questions and improved the answer options of the questions. The revised questionnaire had an acceptable level of face and content validity and readability. Demographic data included age, residence (Urban/Rural), per capita income of the family (<¥4500/¥4500 and ¥9000/>¥9000), occupation (Rural migrant workers/Urban and rural unemployed, unemployed/Industrial workers of Non-agricultural registered permanent residence/Individual business/Business services staff/Civil servants/Senior manager and Middle-level manager in large and medium enterprise/Private entrepreneur/Professionals/Clerks/Students/Others), advanced age for pregnancy(aged 34 years old and above) (YES/NO), chronic diseases (YES/NO), hospital level (Level 3A hospital/Level 2A hospitals/Level 2B hospitals and below), nationality (Han nationality/Minority), only child (Yes/No), husband is the only child (Yes/No), marital status (Unmarried/Marriage/Remarried/Divorced/Widowed). Pregnancy was divided into three trimesters. Education level was categorized as ≤primary school, junior middle school (basic education), ≥a senior high school (including vocational/technical secondary school and junior college), (secondary education) and ≥senior college and university (higher education). CS delivery intention among pregnant women were divided into 3 categories “Normal delivery intention”, “Unsure prior to delivery”, and “Caesarean delivery intention”.

### 2.7. Data Analysis

The data was carefully reviewed prior to entry into the database using EPI Data 3.1 software (The EpiData Association, Odense, Denmark). Data analysis was performed using statistical software (SAS version 9.1; SAS Institute, Cary, NC, USA) after careful data sorting and cleaning. The characteristics of the participants were summarized using either means and standard deviations or frequencies and percentages, and were presented using descriptive analysis (means, standard deviations, and percentages). Chi-square tests were employed for comparisons when appropriate. Ordinal logistic regression analysis [18] was conducted to examine the risk factors of CS delivery intention among expectant mothers.

## 3. Results

### 3.1. Demographic Characteristics of Expectant Mothers

In total 1755 and 590 expectant mothers in their first and second pregnancies were enrolled. 354 (15.1%) intended to deliver by CS and 585 (25.0%) participants were unsure prior to delivery. Furthermore, 156 (8.9%) expectant mothers in their first pregnancy and 198 (33.6%) expectant mothers in their second pregnancy intended to deliver by CS. 346 (15.4%) Han nationality and 8 (8.6%) minority nationality intended to deliver by CS. 42 (14.3%) in the first trimester of pregnancy and 97 (13.8%) in the second trimester of pregnancy, and 215 (15.1%) in the third trimester of pregnancy intended to deliver by CS. 263 (12.5%) participants who were advanced in age for pregnancy, and 91 (37.5%) participants who were not, intended to deliver by CS (see Table 1).

### 3.2. Ordered Multivariate Logistic Regression for Caesarean Delivery Intention

To further investigate the factors that affect the characteristics of women with particular delivery preferences, we chose the following parameters: hospital level, nationality, only child, husband is the only child, marital status, education level, residence, per capita income of the family, career, trimester of pregnancy, advanced maternal age in pregnancy, parity. CS delivery intention (Normal delivery/Not sure/Caesarean delivery) was a dependent variable; we then conducted ordinal logistic regression analysis. In the ordinal logistic regression analysis model, partial regression coefficient (ß) = Estimate. OR = e^ß^. Compared with Han nationality, minority nationalities were less likely to have CS delivery intention (95% CI (−0.9588, −0.0398), *p* = 0.0374). Compared with Expectant Mothers in their First Pregnancy, Expectant Mothers in their Second Pregnancy were more likely to have CS delivery intention (95% CI (0.6467, 1.0584), *p* < 0.0001). Compared with women in early pregnancy, women in late pregnancy were less likely to have CS delivery intention (95% CI (−0.5215, −0.0209), *p* = 0.0327). Women with advanced maternal age in pregnancy were more likely to have CS delivery intention (95% CI (0.4593, 1.0308), *p* < 0.0001) (see Table 2).

## 4. Discussion

In view of the increasing CS rate in mainland China and the rest of the world [2], especially among pregnant women without any medical indications for CS, we investigated the child delivery preference among Chinese pregnant women during the transition of China’s one-child policy. Our study shows that the preference of the natural childbirth accounted for 59.8%, CS 15.09% and “not sure” 24.94%. Consistent with these findings, Liu et al. [19] conducted a similar study among Shanghai pregnant women over last six years and reported that more people preferred vaginal birth (61.2%) than CS (24.7%). However, our study indicated that the proportion of intended CS delivery among second-time mothers is much higher than that of the first timers.

In the ordinal logistic regression analysis model, this study found that compared with expectant mothers in their first pregnancy, expectant mothers in their second pregnancy were more likely to have CS delivery intention. Pregnant women of advanced age were more likely to have CS delivery intention; they may have had more experiences and psychological pressures to undergo a vaginal delivery. Of course, women childbearing for the second time is generally older than women childbearing for the first time, with higher pregnancy risk as well. Advanced maternal age is associated with higher risks of miscarriage, premature birth, stillbirth and higher rates of gestational diabetes mellitus, gestational hypertension and preeclampsia-eclampsia [20,21]. Overweight and obese older pregnant women have a significantly high risk, particularly for stillbirth and preterm delivery [22]. Therefore, it is critically important to provide the appropriate prenatal care (disease detection and prevention) to women who are pregnant for the second time. Effort should also be put to improve the quality of the medical professionals and their continuing education, strengthening the obstetric management, and monitoring the CS criteria. In order to reduce the unnecessary adverse outcomes, effort should be increased to strengthen the monitoring and management of older pregnant women so that reasonable dietary guidance and weight monitoring [23] can be provided, as well as improving health care awareness of physical health and social and psychological wellbeing. The Chinese government implemented the one-child policy nearly 40 years ago, and the CS rate gradually increased. The two-child policy will increase birth rates; this study found that expectant mothers in their second pregnancy were more likely to choose CS delivery. Consequently, the CS delivery rate may further increase. This study provides more important implications for the control of CS deliveries after China’s new universal two-child policy.

An interesting phenomenon in the survey is that the preference for CS among ethnic Han women is higher than that of ethnic minorities. Possible reasons include special phenomena, such as a desire for children to be born on special days named “Auspicious day”, even “Auspicious hours”and thus, choose CS delivery. Women’s awareness of childbirth is also subject to cultural, social norms and expectations, as well as their local medical conditions and the medical, advice they receive [24,25]. Other studies also demonstrated the influence of religions and communities on the attitude to CS [14]. For example, Janevic et al. [26] surveyed the delivery preference of women from different races and birthplaces in New York; after adjusting for multiple risk factors, women’s CS preference differed by race and birthplace. All ethnic groups except East Asian women were at an increased risk of CS delivery; the highest rates were among Hispanic Caribbean women and African American women. Janevic et al. suggested that some potential factors should be further explored, including hospital environment, provider’s bias, and patient preference. Efforts to reduce CS rates should address these disparities. Henderson et al. investigated the quality of obstetrics services for minority groups in England and Wales. They found that compared with whites, minorities have poorer obstetric services, and they argued that these services should be improved [27].

Compared with women in early pregnancy, women at the late stage of pregnancy were less likely to have CS intention. This may be consequent on prenatal pathology (such as malposition, pregnancy-induced hypertension, gestational diabetes, fetal growth restriction, an oversized infant, twins, umbilical cord around the neck and other fetal anomalies) and psychological changes. As they get closer to their due date, pregnant women may be more likely to pay attention to the decision of delivery mode. Women under anxiety and psychological tension are particularly vulnerable to external influences; for example, inpatients could be affected by women who are in labour, as well as by information from the medical staff.

A 2010 report is worthy of mentioning; this re-defined the concept of term infants from 39 to 40 weeks (+6 days) [28]. The report pointed out that infants born less than 39 weeks may not perform optimally in the long run in reading and maths. It was believed that week 37–39 of the pregnancy is a critical development period of fetal brains [28]. Therefore, in the hospital, unless there is a medical exception, it is important for medical staff to nurture the correct delivery attitude, and organize periodic training, and to foster patient’s confidence and a positive attitude. Although CS could effectively reduce the high-risk pregnancy complications and neonatal mortality, unnecessary CS should still be avoided for low-risk deliveries. Of course, patient’s needs and the precondition of mother-baby safety must be taken into account in decision making.

To decrease the C-section rate, we should first decrease the rate of CS through maternal request. Appropriate policies and guidelines should be developed to accomplish this goal [6]. CS delivery rates positively correlate with infant mortality rates among high-income industrialized countries. One cause of this phenomenon is iatrogenic preterm delivery [29]. Global health care professionals should put actions to strengthen the obstetric care and the accurate assessment of CS criteria [9], including providing of options of painless childbirth and education and psychological interventions, increasing of quality of natural delivery services, proper culture and prohibiting of doctors from professional opinions and profit [30].

This study has certain limitations. First, cross-sectional survey data reduced the ability to make direct causal inferences, to explore whether unmeasured factors may better explain the observed relationships we observed, and to determine the direction of causality. Second, the face-to-face survey administration design may convey information bias. Respondents may not have answered the questions truthfully. That said, all questions in the survey were reviewed by a panel of researchers and participants in the pilot study, and thus, the questionnaire was less likely to include items that could be perceived as sensitive by the study participants. During the face-to-face interview, investigators asked questions one by one to assure that respondents would answer seriously. Third, the authors have not actually asked the about the reasons for participants’ choice of delivery, this study mainly focuses on the characteristics of the participants who chose caesarean delivery. Fourth, our study was not exactly nationally representative. The sample consisted of pregnant women in five regions, namely, Chongqing, Chengdu, Zunyi, Liaocheng, and Tianjin, China. Chongqing, Chengdu, and Zunyi are in south China, Liaocheng and Tianjin are in north China.

## 5. Conclusions

Estimated 8.9% of expectant mothers in their first pregnancies and 33.6% of mothers in their second pregnancies intended to deliver by CS. This study provides more important implications for the control of CS deliveries after China’s new universal two-child policy. Any intervention programs to reduce the rate of CS should focus on the Han minority, pregnant women of advanced age, women in early pregnancy and, expectant mothers in their second pregnancy.

## Figures and Tables

**Table 1:** The demographic characteristics of expectant mothers stratified by intended delivery mode.

Variable	Model of Delivery		
	Normal Delivery	Caesarean Delivery	Unsure Prior to Delivery
**Parity**			
Expectant Mothers in their First Pregnancy	1133 (64.56%)	156 (8.89%)	466 (26.55%)
Expectant Mothers in their Second Pregnancy	273 (46.27%)	198 (33.56%)	119 (20.17%)
**Hospital level**			
Level 3A hospital	1066 (58.44%)	270 (14.80%)	488 (26.75%)
Level 2A hospitals	198 (63.67%)	59 (18.97%)	54 (17.36%)
Level 2B hospitals and below	142 (67.62%)	43 (20.48%)	25 (11.90%)
**Nationality**			
Han nationality	1342 (59.59%)	346 (15.36%)	564 (25.04%)
Minority	64 (68.82%)	8 (8.60%)	21 (22.58%)
**Single-child**			
Yes	608 (58.13%)	166 (15.87%)	272 (26.00%)
No	798 (61.43%)	188 (14.47%)	313 (24.10%)
**Husband was single-child**			
Yes	698 (59.51%)	165 (14.07%)	310 (26.43%)
No	708 (60.41%)	189 (16.13%)	275 (23.46%)
**Marital status**			
Unmarried	28 (57.14%)	10 (20.41%)	11 (22.45%)
Primary marriage	1333 (60.45%)	326 (14.78%)	546 (24.76%)
Remarried	35 (50.00%)	14 (20.00%)	21 (30.00%)
Divorced or Widowed	10 (47.62%)	4 (19.05%)	7 (33.33%)
**Education level**			
Basic education	231 (57.46%)	71 (17.66%)	100 (24.88%)
Secondary education	195 (55.08%)	63 (17.80%)	96 (27.12%
Higher education	980 (61.67%)	220 (13.85%)	389 (24.48%)
**Residence**			
Urban	1132 (60.21%)	292 (15.53%)	456 (24.26%)
Rural	274 (58.92%)	62 (13.33%)	129(27.74%)
**The per capita income of the family**			
<¥4500	357 (58.43%)	93 (15.22%)	161 (26.35%)
¥4500 and ¥9000	600 (60.67%)	148 (14.96%)	241 (24.37%)
>¥9000	449 (60.27%)	113 (15.17%)	183 (24.56%)
**Occupation**			
Rural migrant workers	66 (55.93%)	18 (15.25%)	34 (28.81%)
Urban and rural unemployed	334 (60.40%)	84 (15.19%)	135 (24.41%)
Industrial workers of Non-agricultural registered permanent residence	26 (52.00%)	7 (14.00%)	17 (34.00%)
Individual business	110 (55.28%)	33 (16.58%)	56 (28.14%)
Business services staff	94 (60.65%)	19 (12.26%)	42 (27.10%)
Civil servants	250 (62.81%	63 (15.83%)	85 (21.36%)
Senior manager and Middle-level manager in large and medium enterprise	53 (55.21%)	18 (18.75%)	25 (26.04%)
Private entrepreneur	49 (56.32%)	16 (18.39%)	22 (25.29%)
Professionals	159 (65.16%)	30 (12.30%)	55 (22.54%)
Clerk	80 (57.55%)	23 (16.55%)	36 (25.90%)
Students	10 (66.67%)	3 (20.00%)	2 (13.33%)
Others	175 (60.14%)	40 (13.75%)	76 (26.12%)
**Trimester of pregnancy**			
first trimester	159 (54.27%)	42 (14.33%)	92 (31.40%)
second trimester	417 (59.49%)	97 (13.84%)	187 (26.68%)
third trimester	830 (61.44%)	215 (15.91%)	306 (22.65%)
**Advanced age in pregnancy**			
YES	1306 (62.13%)	263 (12.51%)	533 (25.36%)
NO	100 (41.15%)	91 (37.45%)	52 (21.40%)

Notes: Education level was categorized as ≤primary school, junior middle school (basic education), ≥a senior high school (including vocational/technical secondary school and junior college), (secondary education) and ≥senior college and university (higher education).

**Table 2:** Ordered Multivariate Logistic Regression for caesarean delivery intention.

Parameter		Estimate	SE	95% CI		*p*−Value
Intercept1		−1.8751	0.2226	−2.3144	−1.4416	<0.0001
Intercept2		−0.4713	0.2182	−0.9017	−0.0458	0.0308
Nationality	Minority	−0.4863	0.2337	−0.9588	−0.0398	**0.0374**
	Han nationality (ref.)					
Single-child	YES	0.1596	0.0878	−0.0126	0.3318	0.0693
	NO (ref.)					
husband is single-child	YES	0.0459	0.0864	−0.1235	0.2154	0.5955
	NO (ref.)					
Marital status	Remarried	0.1946	0.2905	−0.3896	0.7550	0.5031
	Divorced or Widowed	−0.1378	0.2403	−0.6160	0.3283	0.5664
	Unmarried	0.4069	0.4229	−0.4470	1.2259	0.3359
	Marriage (ref.)					
Education level	Secondary education	0.1466	0.1491	−0.1458	0.4390	0.3256
	Higher education	−0.0757	0.1342	−0.3377	0.1884	0.5724
	Basic education (ref.)					
Residence	Urban	0.0452	0.1166	−0.1822	0.2750	0.6984
	Rural(ref.)					
Per capita income of the family	¥4500 and ¥9000	−0.0266	0.1089	−0.2397	0.1874	0.8070
	>¥9000	−0.0450	0.1210	−0.2821	0.1925	0.7101
	<¥4500 (ref.)					
Occupation	Rural migrant workers	−0.1822	0.2368	−0.6497	0.2797	0.4418
	Urban and rural unemployed	−0.0235	0.1518	−0.3201	0.2754	0.8769
	Industrial workers of Non-agricultural registered permanent residence	0.2405	0.2995	−0.3569	0.8210	0.4220
	Individual business	0.0240	0.1851	−0.3398	0.3863	0.8966
	Business services staff	−0.0892	0.2034	−0.4908	0.3074	0.6610
	civil servants	−0.0516	0.1592	−0.3632	0.2612	0.7459
	Senior manager and Middle-level manager in large and medium enterprise	0.1164	0.2362	−0.3513	0.5760	0.6220
	Private entrepreneur	0.0050	0.2436	−0.4784	0.4783	0.9836
	Professionals	−0.2331	0.1804	−0.5882	0.1195	0.1964
	Clerks	0.0927	0.2079	−0.3176	0.4982	0.6558
	Students	0.0421	0.5717	−1.1665	1.1193	0.9413
	Others (ref.)					
Parity	Expectant Mother of Second Pregnancy	0.8526	0.1050	0.6467	1.0584	**<0.0001**
	Expectant Mother of First Pregnancy (ref.)					
Trimester of pregnancy	Mid-pregnancy women	−0.2656	0.1377	−0.5348	0.0053	0.0537
	Late-stage pregnant women	−0.2726	0.1276	−0.5215	−0.0209	**0.0327**
	Early pregnant women (ref.)					
Elderly pregnancy	YES	0.7454	0.1457	0.4593	1.0308	**<0.0001**
	NO (ref.)					

Note: SE, standard error.
